# A Qualitative Examination of Respondent-Driven Sampling (RDS) Peer Referral Challenges Among Young Transwomen in the San Francisco Bay Area

**DOI:** 10.2196/publichealth.4573

**Published:** 2015-08-25

**Authors:** Sean Arayasirikul, Xiang Cai, Erin C Wilson

**Affiliations:** ^1^ Center for Public Health Population Health Division San Francisco Department of Public Health San Francisco, CA United States; ^2^ Medical Sociology Department of Social and Behavioral Sciences University of California San Francisco San Francisco, CA United States; ^3^ Yale University New Haven, CT United States; ^4^ Department of Epidemiology and Biostatistics University of California San Francisco San Francisco, CA United States

**Keywords:** transgenders, young adults, qualitative research, epidemiology, methodology

## Abstract

**Background:**

Efforts have focused on developing innovative recruitment strategies to engage the most marginalized of populations in public health research. Respondent-driven sampling (RDS) has been found to be an effective sampling strategy for hard-to-reach, hidden populations. Though studies have documented RDS peer referral as challenging, literature contextualizing these challenges is scant and rarely do they discuss the role of Internet technologies.

**Objective:**

The objective of the study was to explore reasons for peer referral challenges in a human immunodeficiency virus (HIV) risk and resilience study among a hidden population of youth, specifically, young transwomen. These findings amplify the unique opportunities Internet technologies bring to public health research and methodology.

**Methods:**

We conducted focused, semistructured, qualitative interviews with 16 young transwomen to investigate the reasons why youth did or did not refer peers to an RDS study for transwomen ages 16-24 in the San Francisco Bay Area. Qualitative interview data were coded and analyzed using grounded theory.

**Results:**

Participants discussed specific barriers and facilitators related to four factors that include study design, study implementation, community characteristics, and individual characteristics, which contributed to RDS peer referral challenges.

**Conclusions:**

Our grounded theory analysis identifies important considerations for future RDS studies with hidden youth populations. Exploring research participants’ experiences is integral in strengthening future epidemiologic research efforts that plan to use RDS to sample and estimate the hidden epidemics among at-risk youth and transgender women. Additionally, Internet technologies and Web-based adaptations offer solutions to traditional RDS peer referral challenges, having the potential to increase accessibility and use among hidden youth populations.

## Introduction

### Accessing Transgender Women

Access to hard-to-reach, hidden populations is often limited, but necessary to characterize epidemics among key populations at risk for human immunodeficiency virus (HIV) [1,2]. However, such populations are often difficult to sample [3]. Respondent-driven sampling (RDS), an adaptation of chain-referral sampling [4], has been used to sample diverse hard-to-reach, hidden populations. Transgender women are a population disproportionately impacted by HIV around the world [5] and are considered a hidden population due to gender-based stigma toward this group. RDS studies have been quite successful in reaching transwomen, individuals who not identify with the gender associated with their assigned male sex at birth, for HIV research [6-9], but gaps in specific subpopulations remain.

Research has emerged finding that younger transwomen are also at high risk for HIV [10], but there have been calls for more rigorous population-based studies to assess local epidemics and determine current risk. Younger people in general are a group particularly difficult to recruit using RDS [11-15]. A RDS-evaluation study comparing field data to population data found that being younger was one of only four factors associated with being underrepresented in the RDS data [15]. RDS for finding hidden youth populations has been even more problematic. For example, it took 12 waves of recruitment to achieve a sample size of 259 young women with multiple sex partners in a South African study [12]. It took over three years to recruit a sample of 450 young men who have sex with men (YMSM) using respondent driven sampling in a recent U.S. study [13]. The only factor significantly related to recruitment success in the YMSM study was having a large network size. Some research has been done to identify ways to overcome these issues and to determine what demographic factors are associated, which may give some insight into what populations may be most challenging [13]. No studies were found in the literature that qualitatively investigated reasons for challenges associated with RDS referral among a hidden youth population.

### Purpose of the Analysis

The purpose of this analysis was to fill a gap in the literature by examining the referral experiences of youth participants in an RDS study. Specifically, we sought to address the following research question, “What barriers and facilitators did young transwomen encounter in RDS peer referral?” We discuss how these findings amplify the unique opportunities Internet technologies bring to public health research and methodology and considerations for future applications of RDS peer referral among hidden youth populations.

## Methods

### The SHINE Study

The SHINE study is the first longitudinal study, to our knowledge, of HIV risk and resilience among young transwomen, ages 16-24 [16], in the San Francisco Bay Area. We conducted six focus groups with participants and confirmed feasibility and acceptability of RDS in this hidden, hard-to-reach population. After nine months of RDS implementation, seeds were not propagating, leading to few peer referrals. In order to boost recruitment, we later incorporated direct referrals from community-based organizations, outreach at events, and online outreach through social networks to identify new seeds until a cohort of 300 individuals were enrolled. Participants were compensated for their participation at each data collection time point (baseline, 6-month, and 12-month) in the amounts of US $50, $70, and $100, respectively. At the end of participants’ baseline visit, study staff explained RDS peer referral procedures. Participants were provided with three referral coupons and earned US $20 for each successful referral.

### Participant Recruitment, Procedures, and Analysis

We conducted focused, semistructured, qualitative interviews with a subsample of 16 participants of the parent study via telephone. Participants were purposively sampled in order to obtain diversity in ability/willingness to provide study referrals to peers in age, race/ethnicity, and socioeconomic status. Participants were not paid. Interviews lasted 10-15 minutes and took place during a time that was most convenient for the participant. The interview guide was iterated in order to maximize coverage of participant experiences through theoretical sampling to reach theoretical saturation [17] and to address the following research question, “What barriers and facilitators did young transwomen encounter in RDS peer referral?” The interview guide assessed the following constructs: friendship networks, social isolation; knowledge, attitudes, motivation, and behavior related to peer referral; and peer referral successes, challenges, and improvements.

Interviews were audio-recorded and transcribed verbatim by the second author. Transcriptions were randomly checked for quality and accuracy against original recordings by the first author. Qualitative interview data were coded and analyzed using grounded theory [17]. The first and second authors independently coded qualitative data, line by line, and together, organized codes into categories to identify specific factors that influenced RDS referrals among participants. The last author oversaw all research procedures and is an expert in qualitative research. The Institutional Review Board (IRB) at the University of California, San Francisco, approved all study procedures. All participants provided written consent (or written assent for those younger than 18 years of age in accordance with a review board waiver of parental consent) to participate in the longitudinal parent study. We obtained IRB approval six months later to approach consented participants in the longitudinal parent study to recruit for this substudy in which verbal consent was approved for and obtained.

## Results

### The Participants

The age range of the 16 participants was 17 to 24 years old, with a mean age of 21.25 years. The majority, (10/16) of the subsample, were young transwomen of color, with 12% (2/16) identifying as African American, 25% (4/16) as Asian/Pacific Islander, 19% (3/16) as Latina, and 6% (1/16) as mixed race. There were (50%) 8/16 participants that reported having completed a high school education or less. There were (50%) 8/16 participants that reported a monthly income of US $0-$500. About a third (n=5) of participants were unable to refer a peer, and two-thirds referred 1 or more peers.

Qualitative data revealed specific barriers and facilitators participants encountered while referring their peers. These factors include study design, study implementation, community characteristics, and individual characteristics to explain participants’ perspectives on RDS peer referral. Table 1 is a qualitative matrix showing how participants’ interviews (in columns) were categorized across factors and specific codes (in rows). Table 1 is organized by the number of peer referrals participants made, ranging from unsuccessful recruiters (or those who referred no peers), to moderately successful recruiters (or those who referred 1-2 peers), and, finally, successful recruiters (or those who referred 3 or more peers). For example, Participant A was an unsuccessful recruiter, who made no peer referrals, and reported that the incentives for peer referrals were inadequate (denoted by the “x” in that row for that column) in the Study design factor. Participant A went on to report that the referral process was confusing and that the community was small (in the Study implementation and Community characteristics factors, respectively). Table 1 presents the qualitative analysis of factors that served as barriers or facilitators to participants’ ability to refer their peers.

**Table 1:** Qualitative matrix of factors and codes by participant and their number of peer referrals.

		Unsuccessful recruiters					Moderately successful recruiters					Successful recruiters					
Number of referrals^a^		0					1				2	3			5		7
Participant		A	B	C	D	E	F	G	H	I	J	K	L	M	N	O	P
Matrix of factors																	
**Study design**																	
	Narrow age eligibility			x	x				x				x		x		
	Inadequate incentives	x		x													x
	Adequate incentives		x		x	x	x	x		x	x	x	x	x		x	
**Study implementation**																	
	Paper coupons-difficult to retain					x		x									
	Paper coupons not effective					x					x						
	Referral process confusing	x	x			x	x		x		x				x		
	Maintaining a close relationship with staff														x	x	x
	Actively following up with peers						x					x	x			x	x
**Community characteristics**																	
	Small community size	x	x	x	x		x	x	x	x	x				x	x	
	Sampling saturation			x	x	x	x	x	x	x	x		x				
	Participation as stigmatizing						x				x						
**Individual characteristics**																	
	Social anxiety and discomfort		x			x	x		x								
	Altruism and reciprocity			x						x	x	x		x	x		x

^a^Each column noted by letter represents one participant.

### Study Design

The study’s eligibility criteria created a challenge for participants who wanted to refer other peers. There were five participants that reported that the study’s age eligibility criterion was too narrow (ages 16-24 years old). This negatively impacted participants’ ability to refer peers who were too old for the study, but who made up a substantial part of their social network. There was one participant that reported little access to social settings where other young transwomen were present. She explained,

I am in the upper limit of the age range for the SHINE study, and most of my friends are [older]. You know, I don’t really hang out with younger trans youth.

Study participant

Participants reported the study’s double incentivizing system as both a facilitator and a barrier for referring peers. The US $20 per enrolled participant referral incentive was seen as a good or adequate amount for 11 participants. Though, three participants expressed that the incentive was inadequate. There was one participant that explained that in relation to the amount of work and time it might take to make a successful referral, the incentive was less motivating.

### Study Implementation

Participants identified the study’s referral coupons, referral training, and the relationship with study staff as important to their ability to recruit their peers. For example, three participants who had referred 5 or more peers noted that maintaining a close relationship with research staff supported their referral efforts. There was one participant that even shared that the rapport she developed with research staff over time motivated her to actively seek out potential participants in their network. This participant said that as a result of rapport with study staff, she sought “to be the best possible referral person ever”. Additionally, good rapport with participants helped participants form a better understanding of the study, its objectives, and eligibility criteria. There was one participant that said,

I definitely felt more comfortable when I was completely brought on board with what was going on and what was it about as well as what to say and how to get people in.

Study participant

Additionally, five participants who successfully referred peers were more likely to report practicing active follow-up with peers they gave coupons to. Types of active follow-up these participants implemented included telephone calls, short message service texts, and accompanying referrals to their study visits. Some took the initiative to schedule appointments with study staff for their peers to ensure their follow through.

For two participants, paper coupons were difficult to retain because they were struggling with residential instability; thus, holding onto paper coupons was challenging. There were two participants that reported that the paper coupon was not an effective referral tool. There was one participant that said,

People disregard most of the coupons nowadays. Just like okay, I will just put it somewhere and forget about it.

Study participant

Additionally, seven participants reported that they were confused about how to refer peers or how important it was to refer; as a result, participants often felt little responsibility to refer peers. There was one participant that said,

I am not going to go to somebody and like ‘Hey, how is it going?’ and just hand them out coupons. It is just a little weird, I think.

Study participant

Another participant said that she “was not particularly sure how much information [she] could give out”. Whereas another participant said,

It wasn’t in mind that I needed to tell them. I feel like I was not so informed about the SHINE Study, and I didn’t think I needed to recruit.

Study participant

### Community Characteristics

Participants identified a number of important community/population-specific factors that impacted their ability to refer peers. Specifically, the size of the community, sampling saturation, and participation in research as stigmatizing, emerged as barriers to successful referrals.

There were 11 participants that reported that the young transwomen population overall was very small, which made it very difficult to refer peers. For those who lived outside the metropolitan areas of San Francisco and Oakland, referrals were particularly difficult because the population was even smaller. There was one participant that explained,

I think a lot of girls who also participated in this study live in different areas of the Bay Area. So if you don’t live in San Francisco or you don’t live in Oakland, your network of girls would be a lot smaller.

Study participant

There were 9 participants that reported that the study had reached a point of sampling saturation, saying that most or all of their friends were already enrolled. There was one participant that commented, “I know a lot of people, but a lot of the people I reached out to have already contacted the SHINE study”. Another participant shared, “the challenge [with referring peers] is finding someone who hasn’t already done the SHINE Study”.

Additionally, two participants expressed the belief that participation in research can be stigmatizing. There was one participant that elaborated,

I think [young transwomen] are vulnerable [because] like the study is like a medical experience, like a mental health experience. I think a lot of trans people have a negative impression toward the medical profession. Or like just a general anxiety about it. Like I don’t know, it might feel like going to the doctor without really meaning to, I guess.

Study participant

These participants were influenced by negative experiences with medical and mental health institutions in the past and a deep concern around protection of one’s privacy.

### Individual Characteristics

At the individual level, a number of participants reported experiencing social discomfort around RDS referrals, while others reported altruism as a motivator for peer referral. There were four participants that expressed that they had feelings of social anxiety and discomfort associated with referring peers. There was one participant that explained,

People stress me out, and I don’t like to talk to people. I don’t get social cues. I don’t know; I am just bad at it. I just like to go home and watch Netflix. It’s just not my personality to try to get people to do things. That is why I am not in sales. That is why I sit in front of a computer and program all day, where I don’t have to talk to people.

Study participant

Participants also reported altruism and reciprocity, which helped to motivate their own participation in the study, and subsequently helped motivate some youth to make peer referrals. There was one participant that said,

[The study] is pretty fascinating. In like since it is specifically for [young] transwomen, I was pretty impressed. So like I think, it was an honor to be a part of it...I would be willing to do it without receiving anything. I don’t really care for it. Because, I like, I feel like the purpose and the goal is more important.

Study participant

There were seven participants that reported that their own participation in this study was motivated by helping the community of young transwomen and they, in turn, would benefit from the impact of the study’s findings.

## Discussion

### Principal Findings

These data identify specific factors that serve as barriers and facilitators to RDS peer referral among young transwomen and suggest important considerations for future RDS studies with hidden youth populations. Contrary to adult RDS studies that found low monetary incentives to be inadequate for generating peer referrals, data in this study found that the majority of participants reported the US $20 incentive as adequate. Previous RDS studies conducted in adult populations have experienced challenges in successfully incentivizing peer referrals [18,19]. An RDS study of HIV risk among international MSM travelers offered a secondary incentive for peer referrals in the amount of US $10 [18]. Participants in this study did not respond to that level of monetary incentivization, which prompted study investigators to instead offer participants a raffle entry for prizes of US $500. Another study found that even at that level of incentivization, raffling large monetary sums was not effective among an adult sample of cannabis users [19]. Finding that this level of incentivization was effective for youth supports future RDS studies with youth populations.

With regard to RDS implementation, participants identified challenges around the use of paper coupons and confusion around the referral process. Methodological developments in RDS studies have expanded to include the use of Web adaptations of RDS, referred to as webRDS, which may help to address these challenges [11,20]. WebRDS uses an Internet portal to assign unique identifiers to participants and enable them to generate electronic coupons and linked email messages, which can be sent to peer referrals [20]. Because few public health studies have utilized webRDS [11,18,21-23], a rigorous exploration of webRDS among hidden youth populations may ameliorate challenges we found associated with the use of paper coupons. There was one study that observed that when presented with the choice, study participants strongly preferred electronic coupons to paper coupons [18]. The success of electronic coupons in this study has even suggested that electronic referrals may enhance random selection of peers if participants were not limited to in-person meetings to transfer paper coupons [18].

Broadly, our data found that youth participants were influenced by research-related, relationship, and interactional factors rather than the adequacy of monetary incentives. For example, we found that participants who maintained close working relationships with research staff were more motivated and, as a result, able to successfully refer peers. There was one study that found that though youth often want the potential benefits of research, they vary in their cognitive ability to understand important research details and procedures [24]. Our findings underscore the importance of providing a supportive environment with multiple engagement opportunities for youth participating in research, similar to other published work calling for increased engagement with youth and adolescent communities to address perceived barriers to participation [25,26].

Related to research participation in general, we found that youth participating in research can be stigmatizing and is important for understanding the use of RDS among hidden populations. This is especially true for research studies with vulnerable and marginalized youth that rely on the assumption that participants be “out” as trans, at the very least to themselves and the researchers [27]. Literature has identified many barriers to research participation among socially disadvantaged groups, such as medical mistrust, fear of authority, stigma, mistreatment, or exploitation; these reasons were especially salient for racial, gender, and sexual minorities [3,28-30]. Moreover, HIV research studies have struggled to sample adolescents due to HIV-related perceived stigma and negative social consequences [27]. WebRDS may create opportunities to address these larger research-related issues, making research more accessible and youth-friendly [11]. WebRDS has been found to address some of these challenges around communicating with potential peer referrals by affording youth the ability to recruit peers through passive or active strategies, using the approach that they prefer most [11]. WebRDS may alleviate the social anxiety and discomfort participants reported related to recruiting peers and possibly aid in protecting their anonymity or the confidentiality of their gender identity [11].

### Limitations

There are a number of limitations to this study. The small size of this subsample limits the generalizability of these results to both the large cohort of the parent study as well as the population. Though purposive sampling was used to generate a range of experiences in recruitment, it is subject to selection bias. The extent to which social desirability bias influenced participants’ discussion with researchers about the quality of the research experience is a possibility. Additionally, these data are constrained by brief one-time interviews centered on a specific topic, RDS peer referral. Despite these limitations, these findings highlight the import in examining the experiences of research participants themselves. Most importantly, these data seek to inform and bolster future epidemiologic research efforts that use RDS to sample and estimate the burgeoning HIV epidemic among at-risk youth and transgender women.

### Conclusions

Our findings identified important considerations for the implementation of RDS in communities of young transwomen. Qualitative data identified specific factors related to study design and implementation and community and individual characteristics that impacted participants’ implementation of RDS peer referrals. Specifically, these findings identify strategies that may strengthen future RDS peer referrals and epidemiologic surveillance methods for sampling young transwomen.

Future research building on the methodology and best practices of RDS implementation is necessary to understand and improve the sampling of vulnerable and hard-to-reach minority youth in public health research. More qualitative studies examining the challenges of RDS peer referral may help to build a larger literature base of RDS best practices. Public health practitioners and researchers can then refer and reflect on these studies to help overcome RDS peer referral challenges they may encounter. Additionally, future studies assessing webRDS in comparison with traditional in-person RDS could reveal important findings about when and for which population Internet technologies play a critical role in reaching.

## Abbreviations

HIV: human immunodeficiency virus
IRB: Institutional Review Board
RDS: respondent-driven sampling
webRDS: Web adaptations of RDS
YMSM: young men who have sex with men
